# Tuning the performances of perovskite solar cells using effective organic molecular hole-transporting materials: a review

**DOI:** 10.3389/fchem.2025.1694198

**Published:** 2025-11-26

**Authors:** Xiu-Wen Zou, Piao Guo, Ying-Ying Zhou, Yi-Ming Luo, Yuan-Yuan Li, Qi-Yuan Yang

**Affiliations:** Guangxi Key Laboratory of Natural Polymer Chemistry and Physics, College of Chemistry and Materials, Nanning Normal University, Nanning, China

**Keywords:** hole-transporting materials, perovskite solar cells, organic molecular material, spiro-OMeTAD, power conversion efficiency

## Abstract

This review discusses the current designs and synthetic procedures for organic small molecules as hole-transporting materials (HTMs) by focusing on their structure–property correlations, short-circuit current density (*J*_sc_), open-circuit voltage (*V*_oc_), fill factor (FF), power conversion efficiency (PCE), and material optimizations. While optimizing non-planar spiro-like compounds, various conjugated aromatic, planar molecules, and even porphyrin metal complexes have been developed and studied for the generation of new HTMs. Heteroatoms like oxygen, sulfur, nitrogen, silicon, and selenium have been proven to be beneficial for the development of more stable and cost-effective HTMs and perovskite solar cells (PSCs). Thus, developing new organic molecules as HTMs or HTM dopants can be considered a viable approach for PSCs. A deeper understanding of the organic small molecular perovskites/HTMs can also provide insights into the design of novel molecular architectures capable of achieving effective and stable PSC systems. Finally, we present the outlook for further developments of conventional PSCs with organic molecular HTMs.

## 1 Introduction

**FIGURE 1:**
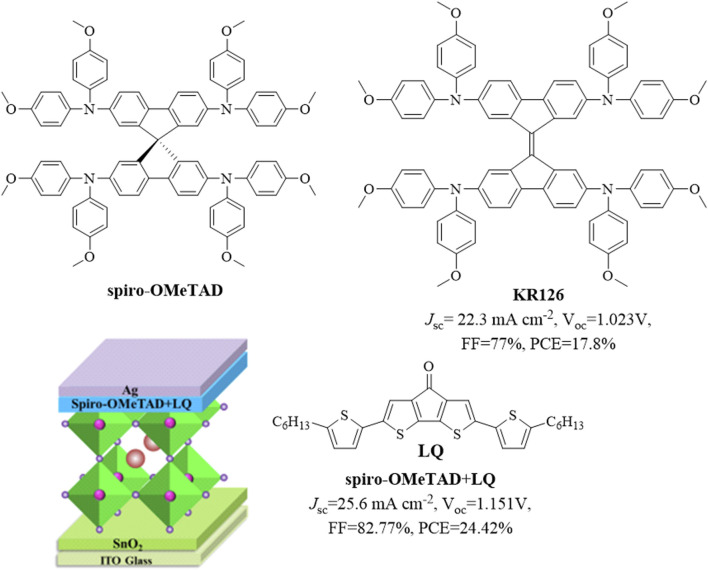
Structures of spiro-OMeTAD, KR216, and LQ-doped spiro-OMeTAD.

Perovskite solar cells (PSCs) were first reported by Kojima et al. (2009) and have rapidly gained popularity in photovoltaic applications since then owing to their unique optical and electrical properties. Perovskite materials can be used as sensitizers for dye-sensitized solar cells, and the highest certified efficiency among solar cells using perovskite materials as absorbing layers has exceeded 26% under iterative development. PSCs have also garnered substantial interest as one of the important classes of third-generation solar cells owing to their excellent power conversion efficiency (PCE) and low-cost manufacturing (Afraj et al., 2025). As one of the important components of PSCs, hole-transporting materials (HTMs) are used to extract and transport photogenerated holes from the perovskite light-absorbing layer to the counter electrode, prevent reverse transport of electrons, as well as protect the perovskite layer from the effects of moisture, oxygen, and metal electrode diffusion in the air. Thus, the performance of the HTM determines the performance of the PSC as well as affects hole transportation and electron–hole recombinations directly. The HTM is one of the key factors in improving the efficiency and stability of a PSC device, and high-performance HTMs are essential for commercializing PSCs. In recent years, high-performance HTMs based on new substituents or doping strategies have been reported. Spiro-OMeTAD is a classic HTM, and 4,4′-dimethoxydiphenylamine-substituted 9,9′-bifluorenylidene (KR216) as a spiro-OMeTAD substitute has been reported to have a PCE of 17.8% (Rakstys et al., 2016). It has also been reported that doping a liquid crystal organic small molecule (LQ) into spiro-OMeTAD could increase its PCE from 21.03% to 24.42% (Lai et al., 2023). The structures of these HTMs are shown in Figure 1.

At present, the HTMs available are mainly inorganic materials and organic molecular materials. Optimizing the molecular structures of organic molecular materials can improve the performances and stabilities of the corresponding PSCs. For instance, a hybrid HTM composed of [2-(9-ethyl-9H-carbazol-3-yl)ethyl]phosphonic acid and strong hole-extraction polymers has been reported to have high efficiency and improved ultraviolet stability (Fei et al., 2024); further, p-i-n PSCs based on π-conjugated unique isomeric selenasumanene-pyridine-based HTMs have been reported to have efficiencies of up to 25.05% (certified at 24.70%) (Azam et al., 2025). These organic molecular materials include organic small molecules, organic polymers, and organic metal complexes. The present review summarizes the impacts of structural changes in organic molecular HTMs on device performances reported in recent years to provide a reference for the development of high-performance HTMs for PSCs by focusing on the optimization of substituent designs for spiro-OMeTAD and additive designs based on antisolvent strategies. The following sections detail the use of organic molecular materials as HTMs and their additives as well as the performance improvements to PSCs thereof.

## 2 Organic molecular materials as HTMs

To promote further development of conventional PSCs with dopant-free organic molecular HTMs, there is a clear need to understand the structure–property correlations of these organic molecules and optimize the perovskite/HTM interface. Considering the structural characteristics of the organic molecules, we will classify them into materials containing spiro ring structures, thiophene structures, imidazole/carbazole structures, other structures, and organometallic complexes for detailed understanding.

### 2.1 Organic molecules containing spiro ring structures

**FIGURE 2:**
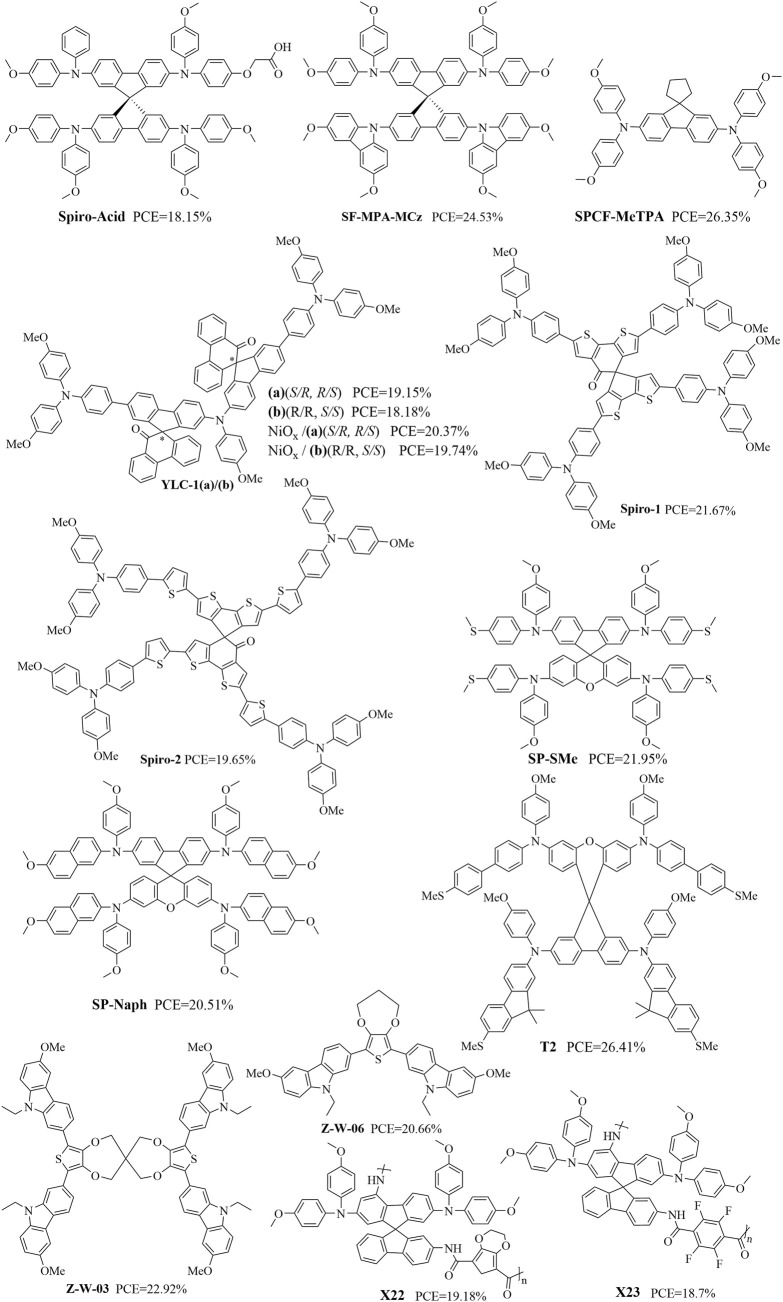
Structural formulas of some representative organic molecular materials containing spiro ring structures.

The structure of an organic molecule has a significant impact on its properties. To further enhance the performances of HTMs, many researchers have attempted to design analogs of spiro-OMeTAD by optimizing the structure of the spiro nucleus molecule. These design optimizations of spiro-HTMs can be roughly divided into three structural categories: end-group optimization (Li et al., 2023; Onozawa-Komatsuzaki et al., 2022; Onozawa-Komatsuzaki et al., 2024; Wang et al., 2024), spiro-core structure regulation (Chiu et al., 2022; Lee et al., 2022a; Liu et al., 2025), and combination of end-group optimization and spiro-core structure regulation (Kumar et al., 2024; Shao et al., 2024; Zhou et al., 2024a).The molecular structures of some representative materials with spiro ring components are shown in Figure 2, and their short-circuit current density (*J*_sc_), open-circuit voltage (*V*_oc_), fill factor (FF), and PCE values are shown in Table 1.

**TABLE 1:** Performance metrics of different perovskite solar cell (PSC) devices with spiro ring hole-transporting materials (HTMs) (*J*_sc_ = short-circuit current density, *V*_oc_ = open-circuit voltage, FF = fill factor, PCE = power conversion efficiency).

HTMs	*J*_sc_ [mA cm^–2^ ]	*V*_oc_ [V]	FF [%]	PCE [%]	References
Spiro-acid	22.2	0.99	82.6	18.15	Li et al. (2023)
SF27 without dopants	21.9	0.92	38	7.6	Onozawa-Komatsuzaki et al. (2022)
SF48 without dopants	22.7	1	72	16.3	Onozawa-Komatsuzaki et al. (2022)
SF67	22.3	1.06	74	18.4	Onozawa-Komatsuzaki et al. (2024)
SF71	23.2	1.06	71	17.3	Onozawa-Komatsuzaki et al. (2022)
SPCF-MeTPA	25.76	1.186	86.3	26.35	Liu et al. (2025)
YLC-1(a)	23.69	1.07	75.56	19.15	Chiu et al. (2022)
YLC-1(b)	23.1	1.06	74.27	18.18	Chiu et al. (2022)
NiO_x_/YLC-1(a)	22.8	1.10	80.93	20.37	Chiu et al. (2022)
NiO_x_/YLC-1(b)	22.78	1.09	79.5	19.74	Chiu et al. (2022)
SP-SMe	24.23	1.16	77.65	21.95	Kumar et al. (2024)
SP-Naph	23.45	1.15	75.73	20.51	Kumar et al. (2024)
T2	26.47	1.175	84.94	26.41	Zhou et al. (2024a)
Z-W-03 doped with Li-TFSI	24.72	1.178	82.5	24.02	Shao et al. (2024)
Dopant-free Z-W-03	23.54	1.115	78.3	20.55	Shao et al. (2024)
Dopant-free Z-W-03/DPB	24.36	1.169	80.5	22.92	Shao et al. (2024)
X22	22.58	1.09	78	19.18	Xu et al. (2024)
X23	22.52	1.05	79	18.70	Xu et al. (2024)

**FIGURE 3:**
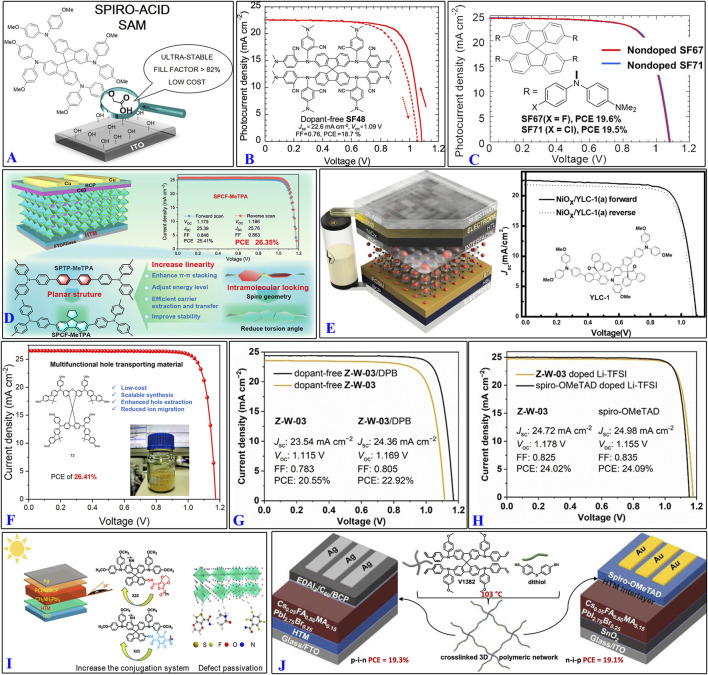
Design highlights of some organic molecules containing spiro ring structures: **(A)** Spiro-Acid; **(B)** SF-48; **(C)** SF-67 and SF-71; **(D)** SPCF-MeTPA; **(E)** YLC-1; **(F)** T2; **(G,H)** Z-W-03; **(I)** X22 and X23; **(J)** V1382. (References: Li et al., 2023; Onozawa-Komatsuzaki et al., 2022; Onozawa-Komatsuzaki et al., 2024; Liu et al., 2025; Chiu et al., 2022; Zhou et al., 2024a; Shao et al., 2024; Xu et al., 2024; Daskeviciute-Geguziene et al., 2023).

Through molecular engineering of the diphenylamine units of spiro-OMeTAD, performances exceeding the PCEs of reference devices based on spiro-OMeTAD have been reported for structures like spiro-acid, SF-MPA-MCz, SF48, SF67, and SF71. The spiro-acid structure was gained by partially replacing one methoxyl group from the diphenylamine units with two oxidaneyl acetic acid groups in the HTM in dopant-free p-i-n hybrid PSCs; the resulting device showed a PCE of 18.15% with ultralow energy loss, which is the highest efficiency among spiro-OMeTAD-based inverted PSCs, along with a remarkable FF of over 82% (Figure 3A) and excellent long-term illumination stability (Li et al., 2023). The SF-MPA-MCz was obtained by partially replacing two diphenylamine units with two rigid carbazole units; here, SF-MPA-MCz exhibited improved thermal stability and hole mobility, suitable energy-level alignment, excellent film morphology, and optimized interfacial contact, all of which contributed to its remarkably high PCE of 24.53% (*V*_oc_ = 1.18 V, *J*_sc_ = 26.24 mA cm^−2^, *FF* = 79.22%) that outperformed the control device based on spiro-OMeTAD (22.95%) (Wang et al., 2024). The SF48, SF67, and SF71 structures were obtained by optimizing the diphenylamine units, which resulted in remarkably high PCEs of 18.7%, 19.6%, and 19.5%, respectively (Figures 3B,C) (Onozawa-Komatsuzaki et al., 2022; Onozawa-Komatsuzaki et al., 2024).

SPCF-MeTPA, YLC-1, spiro-1, and spiro-2 structures have shown remarkably high PCEs of 26.35%, 18.18%–20.37%, 21.67%, and 19.65%, respectively, based on spiro-core structure regulation of spiro-OMeTAD (Chiu et al., 2022; Lee et al., 2022a; Liu et al., 2025) (Figures 3D,E). The structure of SPCF-MeTPA is a rigid conjugated system formed by a fluorene unit and a cyclopentane connected through a spiro carbon center featuring two phenyl rings arranged in a near-planar conformation. The YLC-1 structure was obtained by introducing spiro (fluorene-9,9′-phenanthrene-10′-one) to link the two chirality centers. Spiro-1 and spiro-2 were obtained by introducing two new donor-acceptor-donor (D-A-D)-type spiro-core-based derivatives tethered by four-fold-methoxy-substituted triphenylamine units.

Researchers have also reported synchronous regulation of the spiro-core structure with end-group optimization. For instance, SP-SMe was obtained using spiro[fluorene-9,9-xanthene] (SFX) as the core moiety and replacing the four -OMe groups with methylsulfanyl (-SMe) groups (Kumar et al., 2024; Zhou et al., 2024a), while T2 was obtained using the SFX core moiety and replacing the four diphenylamine units with fluorene units (Figure 3F). The quasiplanar spiro-type Z-W-03 was designed with three carbazole moieties and synthesized as HTMs (Shao et al., 2024), which had remarkably high PCEs of 26.35%, 26.41%, and 20.55%. Upon modification with the hydrophobic dimethylanilinium tetrakis (pentafluorophenyl)borate (DPB), the PCEs of the dopant-free Z-W-03/DPB and Z-W-03 with Li-TFSI dopant increased to 22.92% and 24.02%, respectively (Figures 3G,H). Moreover, polymers like X22, X23, and V1382 were further obtained after end-group optimization, whose PCEs were 19.18%, 18.70, and 19.3%/19.1% (p-i-n/n-i-p PSCs), respectively (Daskeviciute-Geguziene et al., 2023; Xu et al., 2024) (Figures 3I,J). These studies show that it is necessary to retain the classic spiral ring structure while introducing other advantageous structures to improve the performance.

### 2.2 Organic molecules containing thiophene structures

**FIGURE 4:**
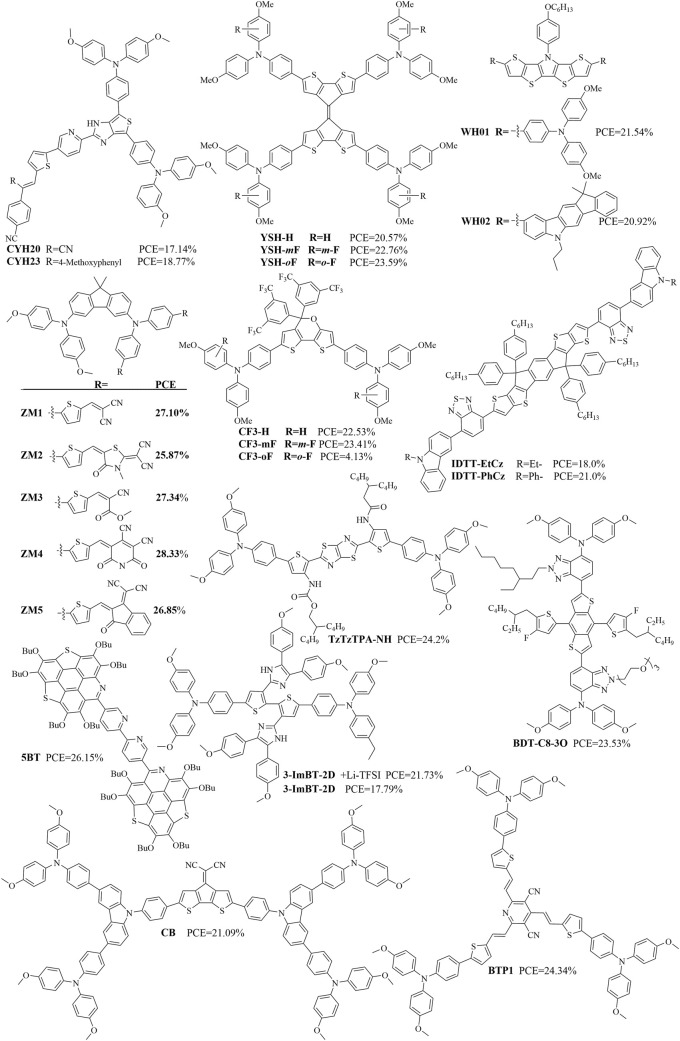
Structures of some organic molecules containing thiophene components (references: Akula et al., 2024; Lee et al., 2023; Wang et al., 2022a; Ali et al., 2023; Lee et al., 2024; Huang et al., 2025; Xie et al., 2025; Cheng et al., 2023; Wan et al., 2025; Xia et al., 2023; Lee et al., 2022b).

**FIGURE 5:**
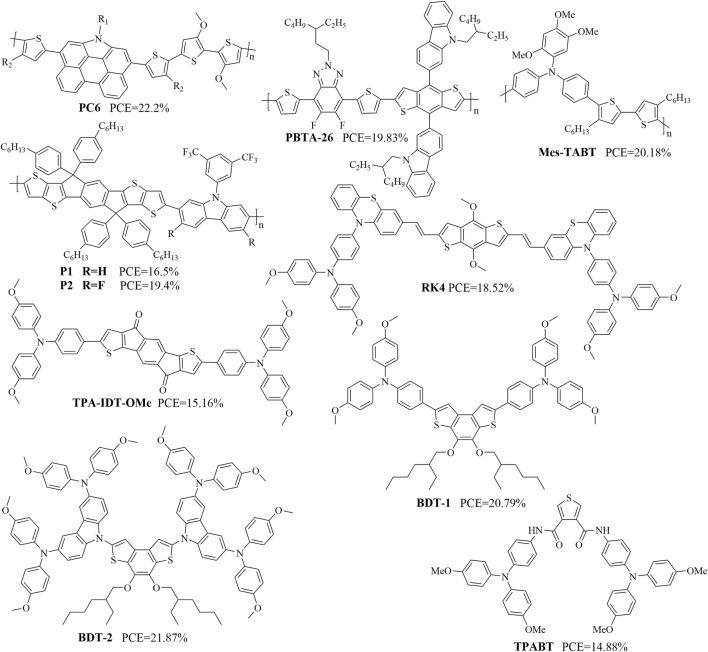
Structures of additional organic molecules containing thiophene components (references: Guo et al., 2021; Yao et al., 2022; Lin et al., 2022; Jia et al., 2022; Gurusamy et al., 2025; Manna et al., 2025; Zhang et al., 2025a; Alkhudhayr et al., 2023).

Organic molecules containing thiophene structures typically exhibit excellent optoelectronic properties and are therefore commonly used as HTMs. Figures 4, 5 show some recently reported HTMs containing thiophene structures, and Table 2 summarizes their *J*_sc_, *V*_oc_, FF, and PCE values. Given the superiority of triphenylamine in spiro-OMeTAD, researchers often retain it when designing HTMs containing thiophene structures, such as CYH23, YSH-oF, YSH-mF, YSH-H, WH01, WH02, ZM1–ZM5, CF3-mF, CF3-oF, and IDTT-PhCz (Akula et al., 2024; Lee et al., 2023; Wang et al., 2022a; Ali et al., 2023; Lee et al., 2024; Huang et al., 2025). The CYH23-based PSC has a high *V*_oc_ of 1.08 V and high *J*_sc_ of 21.78 mA cm^–2^, which have resulted in the highest PCE of 18.77% (Akula et al., 2024). The small-area (0.09 cm^2^) PSCs made from YSH-oF and YSH-mF achieved impressive PCEs of 23.59% and 22.76%, respectively, with negligible hysteresis, in contrast with the PCE of 20.57% for the YSH-H-based device (Lee et al., 2023). PSCs based on both WH01 and WH02 were reported to have PCEs of around 21%, and the optimized PSCs adopting WH01 exhibited a maximum PCE of 21.54% (Wang et al., 2022a). All HTMs designed using ZM1–ZM5 exhibited superior anticipated PCEs (25.87%–28.33%) with higher FF values (89.48%–90.14%) compared to the reference molecule (PCE of 13.22%) (Ali et al., 2023). PSCs employing CF3-mF and CF3-oF showed impressive PCEs of 23.41% and 24.13%, respectively, and the large-area (1.00 cm^2^) PSCs based on CF3-oF achieved a PCE of 22.31% (Lee et al., 2024). CsPbI_3_-based PSCs made with IDTT-PhCz as the dopant-free HTM achieved a maximum PCE of 21.0%, while CsPbI_2_Br-based PSCs using IDTT-PhCz exhibited a maximal PCE of 18.0%; the CsPbI_2_Br/organic tandem solar cell based on IDTT-PhCz was reported to have achieved a high PCE of 25.0% (24.66% certified) (Huang et al., 2025).

**TABLE 2:** Performance metrics of different PSC devices with thiophene-based HTMs (*J*_sc_ = short-circuit current density, *V*_oc_ = open-circuit voltage, FF = fill factor, PCE = power conversion efficiency).

HTMs	*J*_sc_ [mA cm^–2^ ]	*V*_oc_ [V]	FF [%]	PCE [%]	References
CYH20	21.12	1.04	77.81	17.14	Akula et al. (2024)
CYH23	21.78	1.08	79.78	18.77	Akula et al. (2024)
YSH-oF	25.56	1.15	80.24	23.59	Lee et al. (2023)
YSH-mF	25.20	1.14	79.22	22.76	Lee et al. (2023)
YSH-H	24.27	1.10	77.04	20.57	Lee et al. (2023)
WH01	24.37	1.129	78.3	21.54	Wang et al. (2022a)
WH02	23.89	1.121	78.1	20.92	Wang et al. (2022a)
ZM1	25.14	1.2	89.82	27.1	Ali et al. (2023)
ZM2	25.14	1.15	89.48	25.87	Ali et al. (2023)
ZM3	25.14	1.21	89.88	27.34	Ali et al. (2023)
ZM4	25.14	1.25	90.14	28.33	Ali et al. (2023)
ZM5	25.14	1.19	89.75	26.85	Ali et al. (2023)
CF3-H	25.31	1.14	77.39	22.33	Lee et al. (2024)
CF3-mF	25.49	1.16	79.18	23.41	Lee et al. (2024)
CF3-oF	25.51	1.18	80.16	24.13	Lee et al. (2024)
IDTT-EtCz	19.12	1.21	77.8	18	Huang et al. (2025)
IDTT-PhCz	20.30	1.25	82.6	21	Huang et al. (2025)
TzTzTPA-NH	25	1.18	82.1	24.2	Xie et al. (2025)
BDT-C8-3O@3 MC	25.39	1.16	79.87	25.53	Cheng et al. (2023)
5BT	26.05	1.85	84	26.15	Wan et al. (2025)
3-ImBT-2D + Li-TFSI	24.43	1.105	80.5	21.73	Xia et al. (2023)
3-ImBT-2D	23.4	1.022	74.4	17.97	Xia et al. (2023)
3-ImBT-2D@DPB	24.25	1.071	77.4	20.1	Xia et al. (2023)
CB@deposited by spin coating	23.72	1.15	76.1	20.76	Lee et al. (2022b)
CB@deposited by TABC	13.60	1.15	77.7	21.09	Lee et al. (2022b)
BTP1@2 MA	24.95	1.178	82.83	24.34	Yu et al., (2023)
PC6	24.2	1.16	79.6	22.2	Guo et al. (2021)
PBTA-8	22.82	1.142	68.35	17.83	Yao et al. (2022)
PBTA-26	22.87	1.148	75.51	19.83	Yao et al. (2022)
Mes-TABT (1)	23.8	1.15	77	21.3	Lin et al. (2022)
Bu-TABT (2)	23.7	1.04	69	16.9	Lin et al. (2022)
OMe-TABT (3)	19.6	0.74	40	5.8	Lin et al. (2022)
CF_3_CH_2_O-TABT (5)	18.8	0.82	40	6.2	Lin et al. (2022)
P1	20.2	1.09	75	16.5	Jia et al. (2022)
P2	20.7	1.15	82	19.4	Jia et al. (2022)
RK4	24.91	1.06	70.15	18.52	Gurusamy et al. (2025)
TPA-IDT-OMe (1000 lx LED)	157.30	0.89	70.3	30.19	Manna et al. (2025)
TPA-IDT-OMe (1 sun)	23.47	0.95	68.25	15.16	Manna et al. (2025)
BDT-1	24.01	1.125	76.96	20.79	Zhang et al. (2025a)
BDT-2	24	1.127	80.86	21.87	Zhang et al. (2025a)
TPABT	22.59	1.11	59	14.83	Alkhudhayr et al. (2023)
WWC103	23.81	1.09	80.47	20.51	Tingare et al. (2023a)
WWC105	22.71	1.07	81.13	19.74	Tingare et al. (2023a)
WZ103	22.96	1.11	79.2	19.48	Tingare et al. (2025)
H101	23.29	1.09	76	19.18	Wang et al. (2022b)
NY-02	23.71	1.12	76	20.11	Wang et al. (2022b)
NY-03	23.25	1.05	74	17.95	Wang et al. (2022b)
NY-04	23.52	1.09	77	19.65	Wang et al. (2022b)
DPTP-2D	19.60	0.95	37.14	6.93	Afraj et al. (2022)
DPTPo-2D	20.47	1.05	65.25	14.05	Afraj et al. (2022)
DPTP-4D	24.28	1.1	75.54	20.18	Afraj et al. (2022)
F-PBTBDT	23.1	1.13	74.6	19.5	Choi et al. (2022)
Alkyl-PBTBDT	23.1	1.12	74.1	19.2	Choi et al. (2022)

The design of organic molecules containing thiophene structures often involves introduction of other heterocyclic structures based on the thiophene structure to enhance the planarity and optoelectronic properties. One example of such a material is TzTzTPA-NH containing thiophene and thiazole components, which produced a remarkable PCE of 24.2% with good long-term stability; this dopant-free TzTzTPA-NH-based PSC was found to be superior to the doped spiro-OMeTAD-based PSC (Xie et al., 2025). Another example material is BDT-C8-3O containing thiophene and triazole components, whose n-i-p PSCs based on chlorobenzene or the green (natural compound) solvent 3-methylcyclohexanone-processed dopant-free hole transport layer (HTL) showed a maximum PCE of 24.11% (certified of 23.82%) or 23.53% (Cheng et al., 2023). The organic molecular material 5BT contains thiophene and bipyridine structures, and 5BT-modified n-i-p PSCs achieved a maximum PCE of 26.15% (certified at 26.12%) (Wan et al., 2025). The material 3-ImBT-2D contains thiophene and imidazole structures, and PSCs using 3-ImBT-2D as the HTM delivered PCEs of 21.73% with Li-TFSI doping and 17.79% without dopants; further, the Li-TFSI-free device based on 3-ImBT-2D yielded a PCE of 21% after HTM surface modification with the organic p-dopant DPB (Xia et al., 2023). The material CB contains thiophene and carbazole components, and PSCs fabricated via fully scalable processes based on dopant-free CB as the HTM exhibited a PCE of up to 21.09%, which is higher than those of devices based on doped spiro-OMeTAD (14.28%) under the same fabricating conditions (Lee et al., 2022b). BTP1 contains thiophene and bipyridine structures, and 2-methylanisole-processed BTP1-based inverted PSCs with green-solvent-processable HTMs were reported to achieve an impressive PCE of 24.34% (Yu et al., 2023).

The polymer strategy also applies to HTMs containing thiophene structures. A PSC employing PC6, a phenanthrocarbazole-based polymer, as a dopant-free HTM was reported to offer an excellent PCE of 22.2% and significantly improved longevity (Guo et al., 2021). Furthermore, n-i-p PSCs employing the polymer PBTA-26 as a dopant-free HTM exhibited a PCE of 19.83% (Yao et al., 2022). Mes-TABT as a new copolymer containing triarylamine and bithiophene units was shown to function as a HTM for MAPbI_3_-type PSCs; it achieved a maximum PCE of 21.3%, and the storage stability of an unencapsulated device exceeded 1,000 h (Lin et al., 2022). The suitable energy level as well as high hole-extraction ability of P2 was shown to endow n-i-p PSCs with an impressive PCE of 19.4% and high FF of 82%, relative to the limited PCE of 16.5% of the P1-based devices (Jia et al., 2022).

HTMs containing thiophene structures based on various strategies have been reported frequently. For example, devices employing RK1 and RK4 as interfacial layers achieved PCEs of 17.28% and 18.52%, respectively, with RK4 as a standalone HTM reaching a PCE of 16.82% (Gurusamy et al., 2025). The PSCs from dopant-free TPA-IDT-OMe yielded an impressive PCE of 30.19% under indoor light illumination (1,000 lx and 321.6 μW cm^–2^), with high *V*_oc_, *J*_sc_, and FF values of 0.89 V, 157.30 μA cm^–2^, and 70.30%, respectively, which is comparable to the PCEs of doped spiro-OMeTAD-based devices (Manna et al., 2025). PSCs using BDT-2 as the HTM achieved a PCE of 21.87% and outstanding long-term stability, where 92.0% of the initial PCE was retained after 2,400 h of air storage, 85.3% was retained after 550 h of thermal aging, and 80% was retained after 550 h of light soaking (Zhang et al., 2025a). TPABT exhibits a higher bandgap than spiro-OMeTAD and thus more transparent in the visible range of the solar spectrum, leading to lower parasitic absorption losses and increased moisture stability (Alkhudhayr et al., 2023). Solar cells using WWC103 with 2-(1,1-dicyanomethylene)rhodamine as the HTM were reported to exhibit a high *V*_oc_ of 1.09 V and a maximum PCE of over 20.51%; the improved performance of WWC103 over WWC105 (19.74%) was attributed to the new acceptor (Tingare et al., 2023a).

**FIGURE 6:**
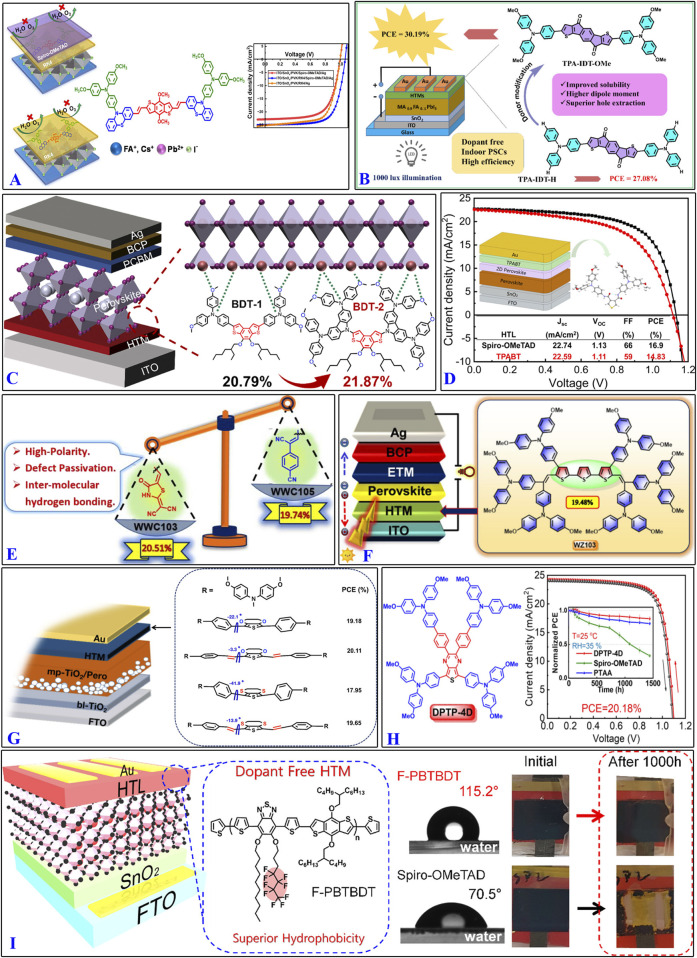
Design highlights of some organic molecular materials containing thiophene structures: **(A)** RK4; **(B)** TPA-IDT-H; **(C)** BDT-1 and BDT-2; **(D)** TPABT; **(E)** WWC103 and WWC105; **(F)** WZ103; **(G)** NY-02 and NY-04; **(H)** DPTP-4D; **(I)** F-PBTBDT. (References: Gurusamy et al., 2025; Manna et al., 2025; Zhang et al., 2025a; Alkhudhayr et al., 2023; Tingare et al., 2023a; Tingare et al., 2025; Wang et al., 2022b; Afraj et al., 2022; Choi et al., 2022).

Given its sulfur-rich terthiophene core, WZ103 has good hole-transporting properties, reduced series resistance, and effective defect passivation that allows a PCE of 19.48% (Tingare et al., 2025). After fabrication into PSCs, devices based on planar NY-02 and NY-04 demonstrate higher PCEs of 20.11% and 19.65% compared to 19.18% and 17.95% obtained with H101 and NY-03, respectively (Wang et al., 2022b). The stable chemical structure of DPTP-4D makes it an effective HTM that delivers a PCE of 20.18% with high environmental, thermal, and light-soaking stabilities than reference HTL materials like doped spiro-OMeTAD and PTAA in planar n-i-p PSCs (Afraj et al., 2022). The PSCs fabricated with F-PBTBDT achieved a high efficiency of 19.5% and maintained 81% of their original efficiency under extremely humid conditions over 1,000 h (Choi et al., 2022). To summarize the above, regardless of the particular strategy used for design optimization of HTMs containing thiophene structures, the goal was always improvement of the rigidity of the molecule. Figure 6 shows some of the design strategies used to obtain organic molecular materials containing thiophene structures.

### 2.3 Organic molecules containing imidazole/carbazole structures

**FIGURE 7:**
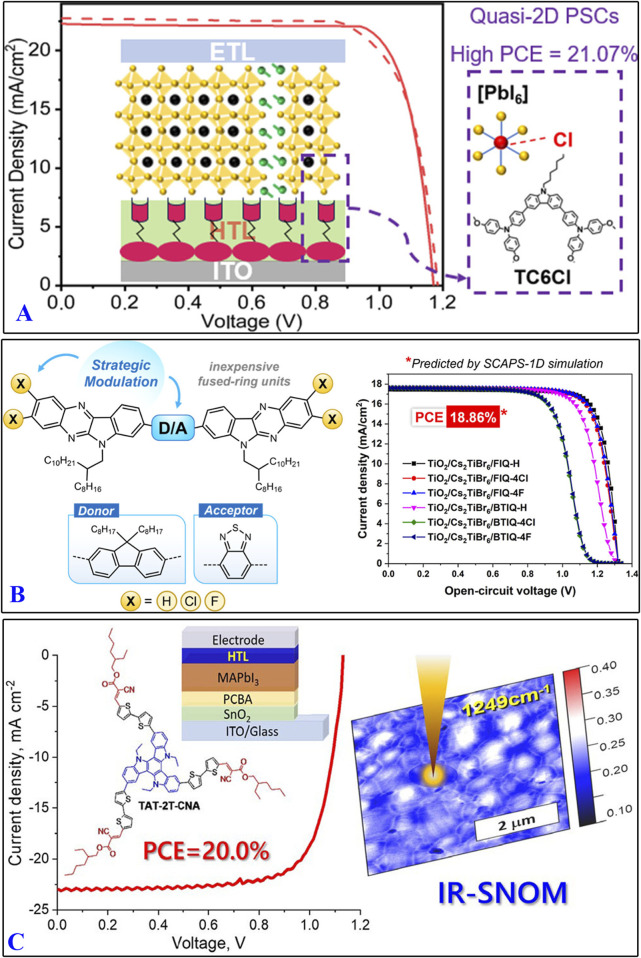
Design highlights of carbazole-based HTMs: **(A)** TC6Cl; **(B)** FIQ-H, FIQ-4Cl, FIQ-4F, BTIQ-H, BTIQ-4Cl, and BTIQ-4F; **(C)** TAT-2T-CNA. (references: Bie et al., 2024; Abid et al., 2025; Latypova et al., 2022).

**FIGURE 8:**
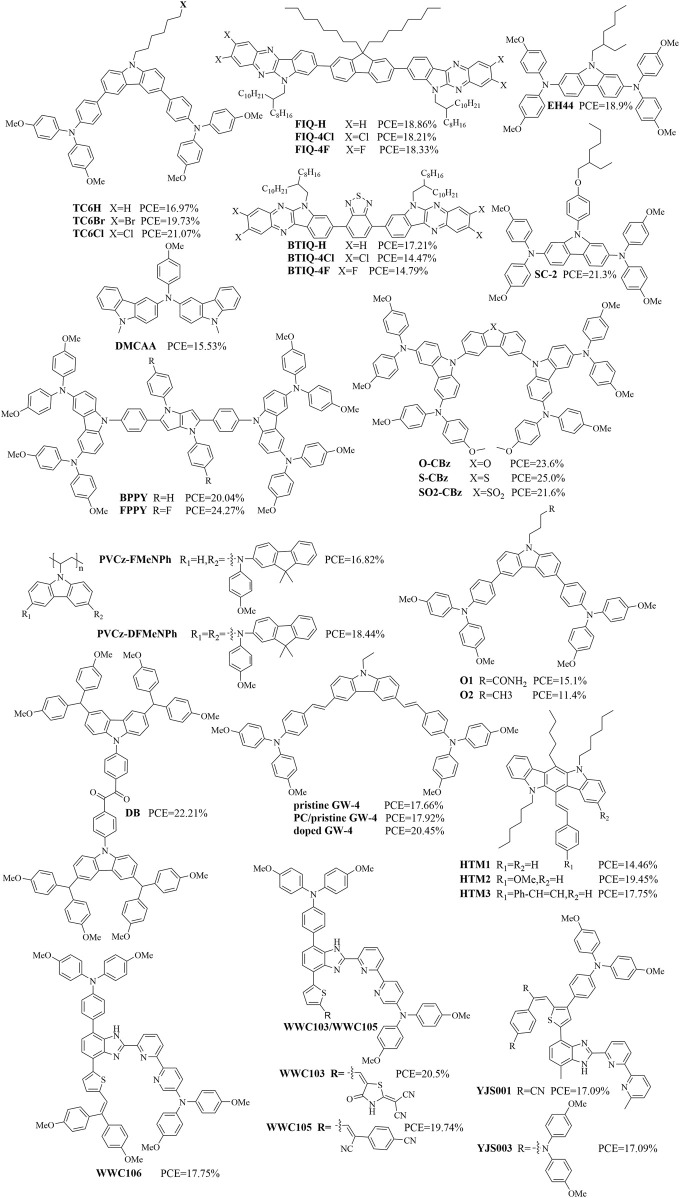
Some organicmolecules containing imidazole/carbazole structures.

Organic molecules containing imidazole/carbazole structures have also been used as HTMs. Figures 7, 8 show some HTMs containing imidazole/carbazole structures and their design strategies reported in recent years, and Table 3 summarizes their *J*_sc_, *V*_oc_, FF, and PCE values. PSCs based on TCnXs (n = 4, 5, 6; X = H, Br, Cl) exhibit significantly enhanced PCEs and stabilities; among these, TC6Cl-based PSCs have achieved a maximum PCE of 21.07% (Bie et al., 2024). Photovoltaic device simulations using SCAPS-1D software have shown promising performances for PSCs incorporating HTMs like FIQ-H, FIQ-4Cl, FIQ-4F, BTIQ-H, BTIQ-4Cl, and BTIQ-4F, with *V*_oc_ values ranging from 1.29 to 1.32 V and predicted PCEs surpassing 18% (Abid et al., 2025). The triazatruxene-based molecule TAT-2T-CNA, which has terminal alkyl cyanoacetate groups and a 2,2′-bithiophene π-conjugated bridge, was shown to have a PCE of 20.1% with negligible hysteresis (Latypova et al., 2022).

**TABLE 3:** Performance metrics of PSC devices with imidazole-/carbazole-based HTMs (*J*_sc_ = short-circuit current density, *V*_oc_ = open-circuit voltage, FF = fill factor, PCE = power conversion efficiency).

HTMs	*J*_sc_ [mA cm^–2^ ]	*V*_oc_ [V]	FF [%]	PCE [%]	References
TC6Cl	22.18	1.17	81.12	21.07	Bie et al. (2024)
FIQ-H	17.62	1.32	81.03	18.86	Abid et al. (2025)
FIQ-4Cl	17.62	1.32	78.2	18.21	Abid et al. (2025)
FIQ-4F	17.62	1.32	78.72	18.33	Abid et al. (2025)
BTIQ-H	17.62	1.32	73.94	17.21	Abid et al. (2025)
BTIQ-4Cl	17.61	1.29	63.73	14.47	Abid et al. (2025)
BTIQ-4F	17.61	1.30	64.56	14.79	Abid et al. (2025)
TAT-2T-DCV	23	1.08	71	17.6	Latypova et al. (2022)
TAT-2T-CNA	23.2	1.12	77	20	Latypova et al. (2022)
TAT-3T-DCV	23.3	1.05	74.1	18.1	Latypova et al. (2022)
BDT-3T-DCV	23.4	1.10	74.2	19	Latypova et al. (2022)
BDT-2T-DCV	23.3	1.08	66.2	16.7	Latypova et al. (2022)
DTS-2T-DCV	23.5	1.06	73.5	17.9	Latypova et al. (2022)
EH44	24.5	1.04	74	18.9	Su et al. (2023)
SC-2	25.1	1.12	75	21.3	Su et al. (2023)
BPPY	22.18	1.13	79.92	20.04	Ravi et al. (2024)
FPPY	25.11	1.18	81.97	24.27	Ravi et al. (2024)
DMCAA	24.75	0.93	67.59	15.53	Keruckas et al. (2023)
O-CBz	25	1.15	82.7	23.6	Yang et al. (2024)
S-CBz	25.3	1.18	83.7	25	Yang et al. (2024)
SO2-CBz	24.7	1.11	78.9	21.6	Yang et al. (2024)
PVCz-DFMeNPh	18.81	1.17	84	18.44	Pan et al. (2022)
PVCz-FMeNPh	18.82	1.16	81	16.82	Pan et al. (2022)
O1	22.6	1.03	64.7	15.1	Wang et al. (2022c)
O2	21.8	0.97	53.8	11.4	Wang et al. (2022c)
SF-MPA-MCz	26.24	1.18	79.22	24.53	Wang et al. (2024)
Pristine GW-4	22.33	1.088	72.7	17.66	Gao et al. (2022)
PC/pristine GW-4	22.81	1.102	71.3	17.92	Gao et al. (2022)
Doped GW-4	22.92	1.125	79.3	20.45	Gao et al. (2022)
DB	23.89	1.15	81	22.21	Tang et al. (2022)
PQ	23.29	1.11	78	20.22	Tang et al. (2022)
HTM1	22.93	1	60.2	14.46	Kim et al. (2022a)
HTM2	24.97	1.07	72.9	19.47	Kim et al. (2022a)
HTM3	24.18	1.06	73.1	18.75	Kim et al. (2022a)
WWC103	23.81	1.09	80.47	20.51	Tingare et al. (2023a)
WWC105	22.71	1.07	81.13	19.74	Tingare et al. (2023a)
WWC106	20.76	1.09	78.9	17.75	Tingare et al. (2023b)
YJS001	22.13	1.054	74.42	17.36	Tingare et al. (2022)
YJS003	23.29	1.093	81.74	20.81	Tingare et al. (2022)

SC-2 is a diphenylamine-substituted molecule that can be used as an efficient and stable HTL in PSCs; it was shown to have a PCE of 21.3%, which is comparable to that of the conventionally doped spiro-OMeTAD (Su et al., 2023). FPPY and BPPY with/without (w/wo) fluorine (F) substitution on the pyrrolo[3,2-b]pyrrole core are connected to the carbazole diphenylamine peripheral end groups, respectively; the F-substituted fused electron-rich pyrrole ring core greatly impacts the molecular surface charge distribution of FPPY, whose PSCs show a best PCE of 24.3% (Ravi et al., 2024). The n-i-p-type PSCs with dopant-free DMCAA as the HTM based on the introduced carbazole compound showed an encouraging PCE of 15.5% (Keruckas et al., 2023). FAPbI3-based PSCs using S-CBz as the HTM achieved a PCE of 25.0%, which is superior to that of spiro-OMeTAD-based PSCs fabricated under the same conditions (23.9%) (Yang et al., 2024). The dopant-free PVCz-DFMeNPh-based inverted quasi-2D PSCs reportedly delivered a PCE of up to 18.44% with negligible hysteresis (Pan et al., 2022). The O1-based n-i-p PSCs displayed enhanced *V*_oc_ (by 60 mV), FF (>11%), and overall PCE (32% increase) values compared to the HB-free O2-based devices (Wang et al., 2022c). These authors have also reported that PSCs employing SF-MPA-MCz show a remarkable PCE of 24.53% (Wang et al., 2024). PSCs with polycarbonate/pristine GW-4 as the HTM have a slightly higher PCE (17.92%) than those with pristine GW-4 (17.66%), and the PCE of doped GW-4-based PSCs (20.45%) was superior to that of spiro-OMeTAD-based PSCs (19.59%) (Gao et al., 2022). Compared to PQ that has a rigid core structure, the benzyl group in DB is flexible with an adjustable molecular configuration, which results in a higher PCE of 22.21% for the DB-based devices than the 20.22% for the PQ-based devices (Tang et al., 2022). Indolo[3,2-b]carbazole-based HTMs (HTM1–3) have been developed for dopant-free PSCs and show different PCEs, namely, 19.45% for the device containing HTM2 > 18.75% for the PSC containing HTM3 > 14.46% for the device containing HTM1 (Kim et al., 2022a). WWC103, WWC105, WWC106, YJS001, and YJS003 are benzimidazole-centered dopant-free HTMs whose PSCs were shown to exhibit PCEs of 20.51%, 19.74%, 17.75%, 17.36%, and 20.81%, respectively (Tingare et al., 2022; Tingare et al., 2023a; Tingare et al., 2023b). From these above works, we note that for HTMs containing imidazole/carbazole structures, the optimization of substituents on N is key to improving the performances of the corresponding PSCs.

### 2.4 Organic molecules containing other structures

**FIGURE 9:**
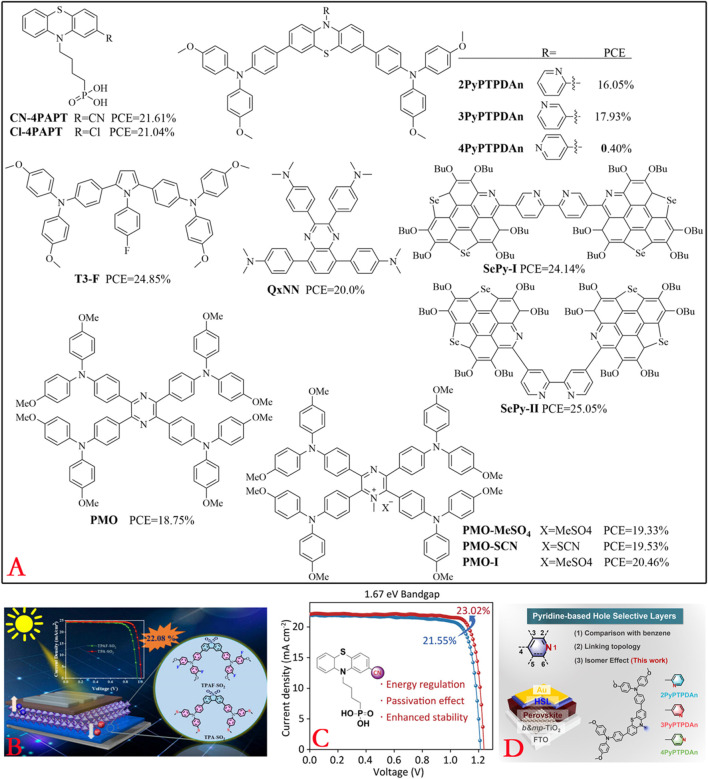
**(A)** Structures of some organic molecules containing heterocyclic components. **(B-D)** Design highlights of TPAF-SO2, CN-4PAPT, 2PyPTPDAn, 3PyPTPDAn, and 4PyPTPDAn (references: Zhou et al., 2024b; Zhang et al., 2025b; Huang et al., 2022).

**FIGURE 10:**
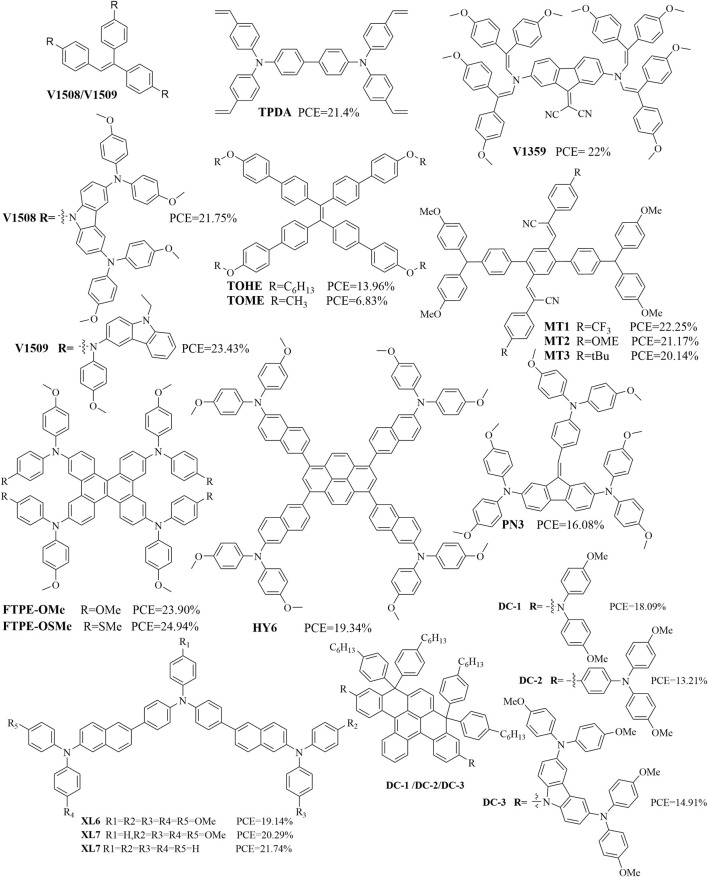
Structures of some organic molecules containing styrene/polyphenyl components.

In addition to spiro ring, thiophene, and imidazole/carbazole structures, the central core of an organic molecular HTM could be a heterocyclic, styrene, or polyphenyl structure. Figures 9, 10 show some HTMs containing heterocyclic, styrene, and polyphenyl structures reported in recent years, and Table 4 summarizes their *J*_sc_, *V*_oc_, FF, and PCE values.

**TABLE 4:** Performance metrics of PSC devices with HTMs composed of other structures (*J*_sc_ = short-circuit current density, *V*_oc_ = open-circuit voltage, FF = fill factor, PCE = power conversion efficiency).

HTMs	*J*_sc_ [mA cm^–2^ ]	*V*_oc_ [V]	FF [%]	PCE [%]	References
TPA-SO2	24.58	1.07	83.95	22.08	Zhou et al. (2024b)
TPAF-SO2	24.31	0.95	79.14	18.42	Zhou et al. (2024b)
Cl-4PAPT	21.02	1.22	81.91	21.04	Zhang et al. (2025b)
CN-4PAPT	22.02	1.24	84.34	23.02	Zhang et al. (2025b)
MeO-2PACz	21.96	1.21	81.1	21.55	Zhang et al. (2025b)
2PyTPDAn	23.36	1.041	66	16.05	Huang et al. (2022)
3PyTPDAn	23.79	1.047	72	17.93	Huang et al. (2022)
4PyTPDAn	3.18	0.966	12.9	0.4	Huang et al. (2022)
T3-F	15.94	1.163	82.37	24.85	Zhou et al. (2023)
NiOx/QxNN	18.4	1.31	82.8	20	Hong et al. (2025)
SePy-I	25.173	1.162	83	24.14	Azam et al. (2025)
SePy-II	25.286	1.176	84	25.05	Azam et al. (2025)
PMO	22.82	1.02	80.12	18.75	Tingare et al. (2023c)
PMO-MeSO4	23.01	1.04	80.67	19.33	Tingare et al. (2023c)
PMO-SCN	22.35	1.07	81.81	19.53	Tingare et al. (2023c)
PMO-I	22.55	1.1	80.96	20.6	Tingare et al. (2023c)
V1509	25.12	1.127	82.75	23.43	Petrulevicius et al. (2024)
V1508	25.84	1.082	77.84	21.75	Petrulevicius et al. (2024)
Cl-TPDA	25.1	1.08	79.1	21.4	Tingare et al. (2023a)
V1359	24.34	1.112	81.13	22.03	Yang et al. (2024)
TOHE	22.1	0.938	70.9	13.96	He et al. (2024a)
TOME	13.45	0.977	50	6.83	He et al. (2024a)
MT1	24.03	1.13	82	22.25	He et al. (2024b)
MT2	23.87	1.11	80	21.17	He et al. (2024b)
MT3	23.41	1.09	79	20.14	He et al. (2024b)
FTPE-OSMe-based PSCs	26.31	1.137	83.37	24.94	Zhang et al. (2024)
HY6	23.00	1.078	78.05	19.34	Liu et al. (2022)
HY5	21.17	1.030	67.84	15.5	Liu et al. (2022)
PN3	20.83	1.046	73.82	16.08	Sánchez et al. (2022)
XL6	23.45	1.09	75.05	19.14	Meng et al. (2025)
XL7	24.46	1.09	75.91	20.29	Meng et al. (2025)
XL8	24.80	1.09	80.19	21.74	Meng et al. (2025)
DC-1	21.89	1.06	78	18.09	Chandrasekaran et al. (2023)
DC-2	17.51	1.02	74	13.21	Chandrasekaran et al. (2023)
DC-3	20.03	1.02	73	14.91	Chandrasekaran et al. (2023)

Heterocyclic HTMs with good performances have been reported in recent literature (Azam et al., 2025; Hong et al., 2025; Huang et al., 2022; Tingare et al., 2023c; Zhang et al., 2025b; Zhou et al., 2023; Zhou et al., 2024b). As shown in Figure 9, organic–inorganic hybrid PSCs based on TPA-SO_2_ as the HTM achieved a PCE as high as 22.08%, whereas the device based on TPAF-SO_2_ achieved a PCE of only 18.42% (Zhou et al., 2024b). The integration of CN-4PAPT as the HTM yielded a certified PCE of 22.66% (Zhang et al., 2025b). The materials 2PyPTPDAn, 3PyPTPDAn, and 4PyPTPDAn have pyridine nitrogen heteroatoms located at the 2, 3, and 4 positions, and PSCs based on these HTMs reportedly delivered PCEs of 16.03%, 17.93%, and 0.40%, respectively (Huang et al., 2022). The triphenylamine-containing pyrrole-based T3-F, T3-H, and T3-OMe HTMs delivered PCEs of 24.85%, 23.58%, and 21.43%, respectively (Zhou et al., 2023). The quinoxaline-based organic molecule QxNN was used as an interlayer between the NiOx HTL and wide-bandgap perovskite in p-i-n PSCs, which remarkably increased the PCE from 17.5% to 20.0% (Hong et al., 2025). Two innovative isomeric selenasumanene-pyridine-based small-molecule HTMs, namely, SePy-I (parallel structured) and SePy-II (orthogonal structured), were recently tailored for p-i-n PSCs and achieved PCEs of 24.14% and 25.05%, respectively (Azam et al., 2025). Furthermore, ionic HTMs like PMO-MeSO_4_, PMO-SCN, and PMO-I having pyrazine as the structural core were incorporated in PSCs to achieve PCEs of 19.33% (*J*_sc_ = 23.01 mA cm^–2^, *V*_oc_ = 1.04 V, FF = 80.67%), 19.53% (*J*_sc_ = 22.35 mA cm^–2^, *V*_oc_ = 1.07 V, FF = 81.81%), and 20.46% (*J*_sc_ = 22.88 mA cm^–2^, *V*_oc_ = 1.10 V, FF = 80.96%), respectively (Tingare et al., 2023c). Comparing the above examples comprehensively, we note that the isomeric selenasumanene-pyridine-based HTMs have more potential for use in PSCs.

Organic molecules with styrene as the core structure could be used as high-performance HTMs (Daskeviciute‐Geguziene et al., 2022; He et al., 2024a; He et al., 2024b; Muniyasamy et al., 2022; Petrulevicius et al., 2024). For instance, V1508 and V1509 based on the triphenylethylene central moiety and carbazole donors as substituents were successfully applied as HTMs in PSCs to achieve PCEs of 21.76% and 23.43%, respectively (Petrulevicius et al., 2024). TPDA is a small molecule with a concise and symmetric structure containing four styrene groups that was designed as a dopant-free HTM for inverted p-i-n PSCs; these inverted devices based on crosslinked TPDA (CL-TPDA) achieved a maximum PCE of 21.4% (He et al., 2024a). The organic molecule V1359 included fluorene and diphenylethenyl enamine units as HTMs, and the corresponding PSCs achieved a PCE of over 22% (Daskeviciute‐Geguziene et al., 2022). Under full exposure to sunlight of AM 1.5G and 100 mW cm^–2^ irradiation, TOHE (with a hexyloxy-substituted phenyl unit) and TOME (with a methoxy-substituted phenyl unit) exhibited device parameters of PCE = 13.96%, *J*_sc_ = 21.00 mA cm^–2^, *V*_oc_ = 0.94 V, FF = 77% and PCE = 6.83%, *J*_sc_ = 13.45 mA cm^–2^, *V*_oc_ = 0.97 V, FF = 52%, respectively (Muniyasamy et al., 2022). Three b-cyanodiarylethene-based X-shaped organic molecules (MT1–3) as HTMs achieved high PCEs of 22.25%, 21.17%, and 20.14% in p-i-n PSC devices (He et al., 2024b).

Organic molecules with polyphenyl cores have also been examined as high-performance HTMs (Chandrasekaran et al., 2023; Liu et al., 2022; Meng et al., 2025; Sánchez et al., 2022; Zhang et al., 2024). A novel dibenzo[g,p]chrysene-based HTM (FTPE-OSMe) with peripheral methoxy and methylthio groups was demonstrated to have a remarkable PCE of 24.94% (certified 24.89%) in PSCs employing Li-TFSI and 4-tert-butylpyridine-doped FTPE-OSMe (Zhang et al., 2024). The HY6-based device (with pyrene core) achieved a PCE of 19.34% that was superior to both HY5-based (with biphenyl core, PCE = 15.50%) and spiro-OMeTAD-based (PCE = 18.33%) devices (Liu et al., 2022). Three organic molecules (PN3, PN2, and T1) containing two and three amino redox centers bridged to a dibenzofulvene backbone were fabricated as HTMs in n-i-p PSCs, which showed PCEs of 16.08%, 15.29%, and 13.53% (Sánchez et al., 2022). Three HTMs based on triphenylamine and N,N-diphenylnaphthalen-2-amines with different numbers of methoxy substituents, namely, XL6, XL7, and XL8, were incorporated in inverted PSCs to yield improved PCEs of 19.14%, 20.29%, and 21.74%, respectively (Meng et al., 2025). Given that polycyclic aromatic hydrocarbons (PAHs) have efficient charge transport and intermolecular interactions, PSCs fabricated with PAH-based HTMs (DC-1, DC-2, and DC-3) reportedly achieved PCEs of 18.09%, 13.21%, and 14.91% (Chandrasekaran et al., 2023). We note that HTMs with styrene or polyphenyl as the central cores still require a triphenylamine structure to achieve good device performances.

### 2.5 Organometallic complexes

**FIGURE 11:**
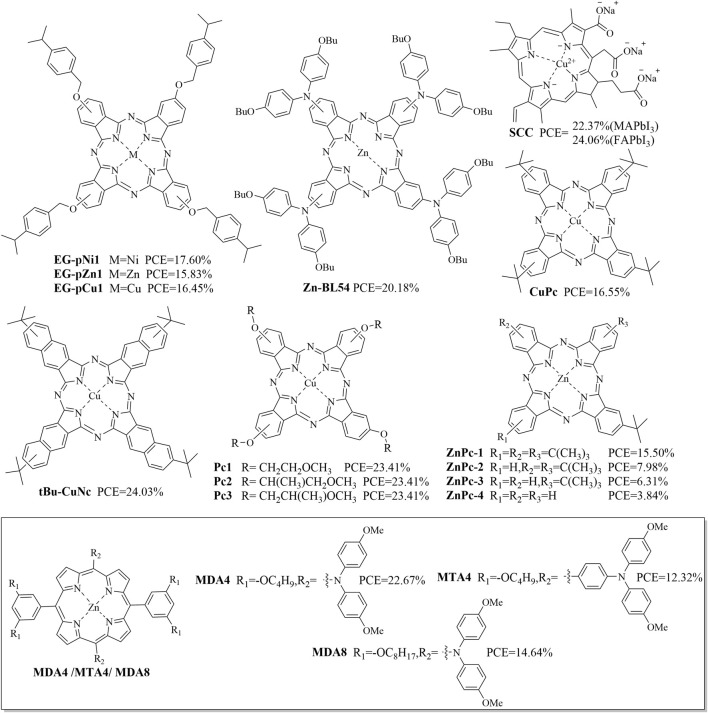
Structures of some organometallic complexes used as HTMs.

Organometallic complexes with multiple fused ring systems have also been used as HTMs owing to their unique delocalized π electron structures. Figure 11 shows some organometallic-complex-based HTMs reported in recent years, and Table 5 summarizes their *J*_sc_, *V*_oc_, FF, and PCE values.

**TABLE 5:** Performance metrics of PSC devices with organometallic complexes as HTMs (*J*_sc_ = short-circuit current density, *V*_oc_ = open-circuit voltage, FF = fill factor, PCE = power conversion efficiency).

HTMs	*J*_sc_ [mA cm^–2^ ]	*V*_oc_ [V]	FF [%]	PCE [%]	References
EG-pNi1	22.91	1.04	73	17.60	Kong et al. (2023)
EG-pZn1	22.61	1.03	67	15.83	Kong et al. (2023)
EG-pCu1	22.87	1.01	71	16.45	Kong et al. (2023)
Zn-BL54	23.73	1.081	78	20	Klipfel et al. (2022)
Cu-BL57	23.66	0.85	78	15.68	Klipfel et al. (2022)
Zn-BL57	23.19	0.88	73	14.89	Klipfel et al. (2022)
Cu-BL61	22.02	0.886	76	14.82	Klipfel et al. (2022)
PTAA/SCC/FAPbI3	25.83	1.16	80.28	24.06	Tian et al. (2024)
CuPc	23.84	0.96	72.55	16.55	Kim et al. (2022b)
tBu-CuNc	25.79	1.15	81	24.03	Qiang et al. (2024)
tBu-CuPc	25.3	1.14	77	22.34	Qiang et al. (2024)
Pc3	25.37	1.14	80.96	23.41	Xiao et al. (2024)
ZnPc-1	24.269	0.932	68.46	15.50	Gassara et al. (2024)
ZnPc-2	18.55	0.772	55.65	7.98	Gassara et al. (2024)
ZnPc-3	17.64	0.704	50.72	6.31	Gassara et al. (2024)
ZnPc-4	12.93	0.658	45.18	3.84	Gassara et al. (2024)
MDA4	24.56	1.135	81.3	22.67	Mai et al. (2022)
MTA4	23.5	0.979	53.52	12.32	Mai et al. (2022)
MDA8	23.5	0.959	64.96	14.64	Mai et al. (2022)

Three dopant-free HTMs based on peripheral 4-(isopropylbenzyl)oxy-substituted phthalocyanines with different core metals (EG-pZn1, EG-pCu1, and EG-pNi1) were shown to produce PCEs of 15.83%, 16.45%, and 17.60% with hysteresis-free characteristics (Kong et al., 2023). Diarylamine-substituted metal phthalocyanines (MPCs, where M = Zn(II) or Cu(II)) functionalized with either linear or branched alkoxy chains have been evaluated as HTMs in PSCs; among these, Zn-BL54 (featuring four n-butoxy side chains) exhibited the highest PCE of 20.18% (Klipfel et al., 2022). The PCEs of inverted PSCs with the SCC modification reportedly increased to 22.37% (from 20.58%) based on MAPbI_3_ and to 24.06% (from 21.54%) based on FAPbI_3_ (Tian et al., 2024). The PCE of CuPc-based PSCs was 16.6%, which could be improved to more than 21% by optimizing the interfacial properties of poly(methyl methacrylate) and perovskite (Kim et al., 2022b); further, PSCs employing tBu-CuNc as the HTM afforded a higher PCE (24.03%) than devices based on CuPc after the structural change from the Pc to Nc core (Qiang et al., 2024).

In n-i-p-type PSC devices, phthalocyanine-based HTMs (Pc1-Pc3) have been shown to achieve PCEs of 22.35% (*J*_sc_ = 25.19 mA cm^–2^, *V*_oc_ = 1.13 V, FF = 78.33%), 21.91% (*J*_sc_ = 25.53 mA cm^–2^, *V*_oc_ = 1.12 V, FF = 81.81%), and 23.41% (*J*_sc_ = 25.37 mA cm^–2^, *V*_oc_ = 1.14 V, FF = 80.96%) (Xiao et al., 2024). HTMs featuring zinc phthalocyanine (ZnPc 1–4) as the central core registered PCEs of 15.50% (*J*_sc_ = 24.269 mA cm^–2^, *V*_oc_ = 0.9329 V, FF = 68.46%), 7.98% (*J*_sc_ = 18.55 mA cm^–2^, *V*_oc_ = 0.9085 V, FF = 55.65%), 6.31% (*J*_sc_ = 17.64 mA cm^–2^, *V*_oc_ = 0.7048 V, FF = 50.72%), and 3.84% (*J*_sc_ = 12.93 mA cm^–2^, *V*_oc_ = 0.658 V, FF = 45.18%) (Gassara et al., 2024). Zn^II^ porphyrin has been used as an effective HTM to fabricate non-spiro PSCs; among these porphyrin HTMs, the MDA4-based PSC delivered the highest PCE of 22.67% (Mai et al., 2022). To summarize the above, most of the organometallic complexes used as HTMs have a transition metal atom at the center of the porphyrin ring.

## 3 Organic molecular materials as HTM additives

**FIGURE 12:**
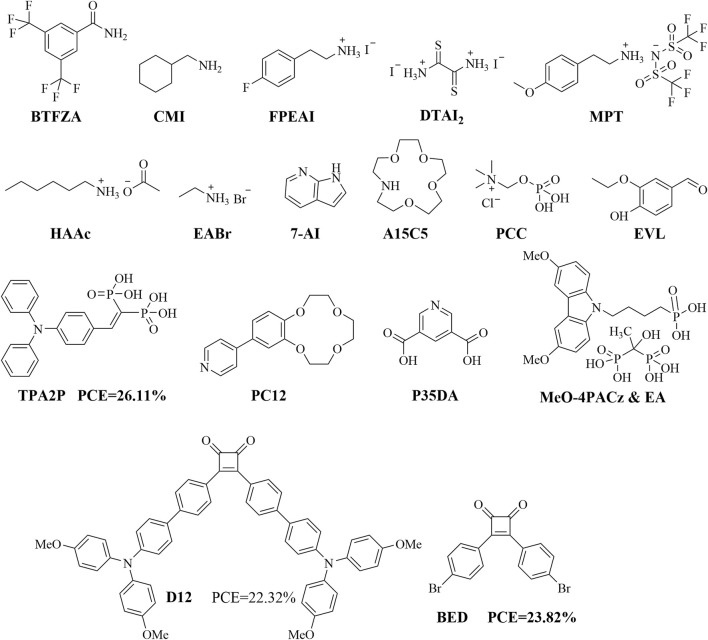
Structures of some organic molecules used as HTM additives.

By ensuring the original optoelectronic performances of HTMs, some additives could be designed and doped into the HTMs to improve their performances in PSCs through the self-assembled monolayer (SAM) and green solvent additive strategies. Figure 12 shows some HTM additives reported in recent years. Through the solvent strategy, BTFZA-modified PSCs exhibited significantly increased PCEs of 20% (Ma et al., 2023). The best-performing device that underwent the double-sided CMI treatment achieved a PCE of 20.66% (Zhou et al., 2025). The interface passivation of FPEAI resulted in PSCs with a remarkable PCE of 19.07%, an enhanced *V*_oc_ of 1.30 V, and FF of 77.8% (Yu et al., 2022). The dithiooxamide iodide (DTAI2) additive was shown to increase the PCE from 18.58% to 21.06% (He et al., 2022). PSCs and mini modules with MPT as the HTM dopant showed increases in their PCEs from 24.38% to 25.52% and from 19.80% to 21.01%, respectively (Li et al., 2025a). The best HAAc-passivated device reached an efficiency of up to 25.06% (Xu et al., 2025). All photovoltaic parameters of inverted PSCs could be improved by introducing EABr, and their PCE increased from 20.41% to 21.06% (Ren et al., 2022). PSCs modified with 7-azaindole (7-AI) achieved PCE increases from 23.27% to 24.63%, with greatly improved stabilities of the encapsulated devices (Han et al., 2025). The A15C5-modulated PSC achieved an impressive PCE of 24.13% along with excellent humidity, light, and thermal stabilities (Chen et al., 2024). The PSC device based on the PCC-modified SnO_2_ showed a PCE of up to 24.23% upon optimization (Yuan et al., 2024). The organic molecule 3-ethoxy-4-hydroxybenzadehyde (EVL) was employed to passivate surface defects on perovskite films, which resulted in a significant improvement of the PCE from 21.9% to 24.1% (for 6 mg mL^–1^ of EVL passivation) (Lu et al., 2023). TPA2P-based inverted PSCs reportedly achieved a high PCE of 26.11% with an exceptional FF of 85.03% (Yuan et al., 2025). PC12 could be applied at the interface between the perovskite layer and HTL as well as in the perovskite precursor solution to promote a PCE of 24.8% (Yu et al., 2024). The devices based on NiOx/P35DA exhibit high PCEs of 24.05% and 21.48% for the 1.56 and 1.68 eV PSCs, respectively (Ge et al., 2025). By combining the SAM and green solvent additive strategies, etidronic acid (EA) was used as a postdeposition micromolecule for filling the SAM(MeO-4PACz) interface, which increased the PCE from 20.08% to 24.42% (Li et al., 2025b). In summary, most of the SAM or green solvent HTM additives reported in literature are nitrogen-containing organic molecules that are more conducive for achieving higher PCEs.

## 4 Conclusion and outlook

We conclude from the above discussion that organic small molecules can be used as HTMs to significantly improve the optoelectronic performances of PSCs. Some of the new structural HTMs have reportedly good optoelectronic properties: PSCs containing BED achieved a PCE of 23.82% with greatly improved stabilities of the unencapsulated devices (Niu et al., 2022); D12-based PSCs show a high PCE of 22.32% (Liu et al., 2023); BPZ23 is a new transition-metal-based complex that was used as a dopant-free HTM in inverted PSCs to obtain a 2.14% increase in PCE than its non-metal counterpart BP21 (Tingare et al., 2024). Thus, only effective organic molecular HTMs can tune the performances of PSCs. The following strategies may be adopted to obtain the most effective organic molecular HTMs for PSCs: (a) design analogs of spiro-OMeTAD by optimizing the structure of the spiro nucleus molecule, including end-group optimization, spiro-core structure regulation, and a combination of end-group optimization and spiro-core structure regulation; (b) retain (or optimize) the triphenylamine structure or introduce other heterocyclic structures for organic HTMs containing thiophene structures; (c) optimize the end groups for organic HTMs containing imidazole/carbazole structures; (d) use other heterocyclic or styrene or polyphenyl components as the central core to design novel organic molecular HTMs; (e) transform organic ligands into organometallic complexes for HTMs; (f) design novel organic molecular HTM additives or combine with other HTMs via the hybrid doping strategy.

Despite the advantages of using organic molecular HTMs in PSCs, there are certain limitations that must be addressed. For example, compared to inorganic materials, organic molecular HTMs require expensive organic synthesis catalysts and are difficult to mass produce; this may result in significant production expenses for PSCs. Appropriate synthesis methods and dosage control are also necessary for integrating organic molecular HTMs in PSCs; in this regard, it might be essential to combine multiple new technologies or processes to optimize the organic molecular HTMs or even optimize the PSCs by integrating solvent molecules or additives. Achieving high performances of PSCs depend on the stabilities of the organic molecular HTMs; although these HTMs may be temporarily stable, it is necessary to determine how they might affect the PSCs and ultimately their performances. Research efforts are also needed to ensure that the use of organic molecular HTMs would not negatively influence the robustness, stabilities, and lifespans of PSCs. Given that the inverse design of tailored organic molecules for new HTMs holds enormous potential (Wu et al., 2024), organic molecular HTMs are expected to allow PSCs to achieve high PCEs in the future.
